# Antibody degradation in tobacco plants: a predominantly apoplastic process

**DOI:** 10.1186/1472-6750-11-128

**Published:** 2011-12-30

**Authors:** Verena K Hehle, Matthew J Paul, Pascal M Drake, Julian KC Ma, Craig J van Dolleweerd

**Affiliations:** 1 Molecular Immunology Unit, Division of Clinical Sciences, St George's, University of London, Cranmer Terrace, London SW17 0RE, UK

## Abstract

**Background:**

Interest in using plants for production of recombinant proteins such as monoclonal antibodies is growing, but proteolytic degradation, leading to a loss of functionality and complications in downstream purification, is still a serious problem.

**Results:**

In this study, we investigated the dynamics of the assembly and breakdown of a human IgG_1_κ antibody expressed in plants. Initial studies in a human IgG transgenic plant line suggested that IgG fragments were present prior to extraction. Indeed, when the proteolytic activity of non-transgenic *Nicotiana tabacum *leaf extracts was tested against a human IgG1 substrate, little activity was detectable in extraction buffers with pH > 5. Significant degradation was only observed when the plant extract was buffered below pH 5, but this proteolysis could be abrogated by addition of protease inhibitors. Pulse-chase analysis of IgG MAb transgenic plants also demonstrated that IgG assembly intermediates are present intracellularly and are not secreted, and indicates that the majority of proteolytic degradation occurs following secretion into the apoplastic space.

**Conclusions:**

The results provide evidence that proteolytic fragments derived from antibodies of the IgG subtype expressed in tobacco plants do not accumulate within the cell, and are instead likely to occur in the apoplastic space. Furthermore, any proteolytic activity due to the release of proteases from subcellular compartments during tissue disruption and extraction is not a major consideration under most commonly used extraction conditions.

## Background

Plants are being developed as a manufacturing platform for a range of pharmaceutical proteins such as vaccines, hormones and antibodies. They are attractive for a number of reasons, including low production costs, the ability to assemble and modify multimeric proteins such as monoclonal antibodies (MAbs) and the ease of scalability. However, heterologous (plant-expressed) proteins often face significant yield losses due to proteolytic breakdown, which has widely been thought to be related to tissue homogenisation and protein extraction. There are many reports in the literature of degradation patterns manifesting as small fragments in gels and immunoblotting analyses [1-3]. Depending on the primary sequence of the heterologous protein, the number of susceptible sites and their accessibility to plant specific proteases, plant-expressed proteins may undergo complete proteolysis or partial trimming [4]. Antibodies are often subject to a significant degree of breakdown with between two to five major species (between M_r _40K and M_r _160K) being reported under non-reducing conditions for different antibodies expressed in *Nicotiana tabacum *leaves [1-3,5-7]. As well as affecting the final yield of target proteins, degradation results in a heterogeneous mixture of recombinant proteins which may alter overall biological activity as well as complicating purification processes [8].

Plants produce proteases for a variety of reasons. Proteases are involved in classical biological processes such as plant development, disease resistance, and nutrient remobilisation for reproductive processes [9,10]. In addition, the timing and levels of protease expression can be viewed as markers for the senescence state of plants [11]. Over 800 proteases are encoded within the genome of *Arabidopsis *[10] and expressed in various tissues and organelles. Proteases are abundant in various subcellular compartments, including the vacuole [11] and the apoplast [10], the default destination for antibodies targeted to the secretory pathway [12,13].

It is widely believed that *ex vivo *degradation of the antibody occurs during the extraction process, as a result of proteases released during tissue and cell disruption [14,15] and several strategies have been used to minimise this effect [15-17]. Most commonly, protease inhibitors are added to extraction buffers but these are expensive and therefore not economically viable for extraction at large scale. Other methods to prevent degradation of recombinant proteins have been proposed. Attempts to identify and knock out major protease families have met with limited success [10]. Alternative approaches include confining expression of proteins to selected cell compartments [18-20], targeting transgene expression to tissues with low metabolic turnover [21,22], co-expression of a specific recombinant protease inhibitor [15,23], or by fusion to stabilising protein domains [24]. These are complicated by the fact that targeted proteases are often important for plant development, the broad spectrum of potential protease targets and compromises resulting from alternative *in planta *targeting strategies.

The objective of this study was to determine whether antibody degradation in transgenic plants is predominantly an intracellular or extracellular process and to identify whether processes involved in tissue disruption and protein extraction are indeed major contributors to proteolytic degradation of antibodies.

## Results

### The proteolytic degradation pattern of recombinant MAb 2G12 extracted from *N. tabacum *is not significantly affected by protease inhibition

**Figure 1:**
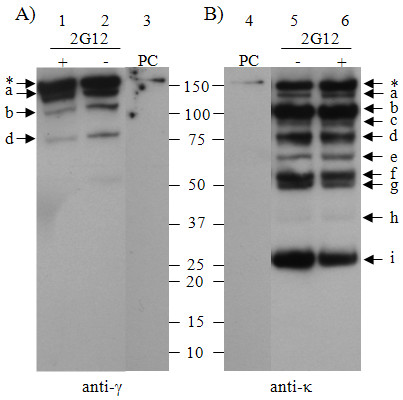
**Western blot analysis of transgenic *N. tabacum *plants expressing MAb 2G12**. Leaf discs from 2G12-expressing plants were extracted in either PBS (-) or PBS supplemented with protease inhibitor cocktail (+) and incubated at room temperature for 24 hours. Proteins were separated by 4-15% SDS-PAGE under non-reducing conditions. The proteins were blotted to nitrocellulose membranes and probed with either anti-human IgG Fc-specific antiserum (Panel A) or anti-human κ antiserum (Panel B). The positive control (PC) consists of the human IgG1κ (1 ng/lane). The asterisk indicates the fully assembled 2G12 antibody; lower case letters (a to i) indicate antibody fragments.

Leaf samples from transgenic *N. tabacum *plants expressing the human IgG_1_κ MAb 2G12 were extracted in PBS (pH 7.4) in the presence or absence of a cocktail of protease inhibitors (Roche Protease Inhibitor Cocktail supplemented with pepstatin A). Analysis was by western blotting under non-reducing conditions (Figure 1), using IgG_1 _heavy chain-specific antiserum (Panel A) or light chain-specific antiserum (Panel B) for detection. A human IgG_1_κ MAb, used as a positive control (PC), demonstrates a predominant band at M_r _150K (indicated with an asterisk) corresponding to fully assembled antibody. In the plant samples, the corresponding major band was always detected. A band at a slightly lower molecular weight (M_r _≈140K) was also detected in both anti-gamma and anti-kappa blots (Figure 1, Panels A and B; labelled a). Together with band b (M_r _100K) and band d (M_r _75K), these were the only major, lower molecular weight species detectable in the anti-gamma blot. In comparison, at least nine bands of lower molecular weight were observed in the anti-kappa blot (Figure 1, Panel B) labelled (a-i). Since some of these bands have molecular weights greater than M_r _25K (the expected size for free light chain), the presence of heavy chain fragments is implied, even though no corresponding fragments are observed in the anti-gamma blot. This was a consistent finding and was also observed when several other human gamma chain-specific antisera were used. Inclusion of a cocktail of protease inhibitors in the extraction buffer did not significantly affect the intensity of the M_r _150K band, or the pattern of smaller bands on the western blots (compare lane 1 with lane 2 and lane 5 with lane 6).

**Figure 2:**
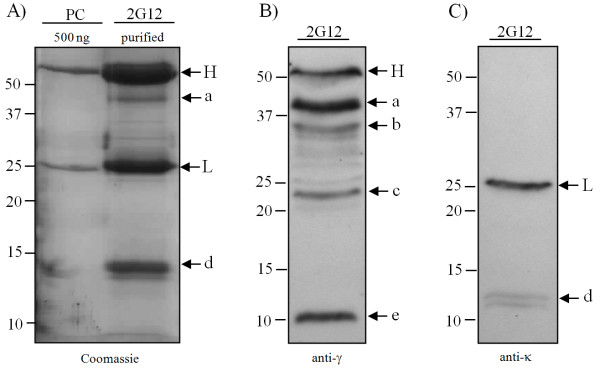
**Analysis, under reducing conditions, of purified 2G12**. 2G12 and antibody fragments from plant extracts were purified using Protein A/G affinity chromatography. Samples were reduced and separated by SDS-PAGE. In Panel A, separated proteins (15% gel) were detected with Coomassie staining. The positive control (PC) consists of 500 ng/lane of human IgG_1_κ. The positions of free heavy and light chains are indicated by H and L, respectively. In Panels B and C, separated proteins (4-15% gradient gels) were blotted to nitrocellulose membranes and probed with either human Fc-specific antiserum (Panel B) or anti-kappa antiserum (Panel C). The positions of the free heavy (H) and light (L) chains are indicated, while the lower molecular weight species labelled a to e indicate antibody fragments.

Protein A/G affinity chromatography was used to purify the antibody mixture (i.e., both fully assembled and lower molecular weight fragments) from 2G12 extracts. These purified proteins were analysed by Coomassie staining of SDS-acrylamide gels under reducing conditions (Figure 2, Panel A). This figure shows that the fully assembled 2G12 antibody and the co-purified lower molecular weight species observed in Figure 1, Panels A and B resolve into two major bands, one of apparent molecular weight M_r _≈ 55K and the other with M_r _≈ 25K (labelled H and L, respectively). The relative molecular weights of these two species, particularly when compared with the free heavy and light chains in the reduced positive control (PC) provide evidence that these 2G12 plant-derived bands are free heavy and free light chains. This is supported by their immunoreactivity with human Fc fragment-specific antiserum (Figure 2, H in Panel B) or human kappa chain-specific antiserum (Figure 2, L in Panel C). In addition, five breakdown fragments (labelled a, b, c, d and e) are also observed. Two of these fragments (a and d) appear to correspond to bands seen in the Coomassie-stained gel. Fragments b (M_r _≈ 35K), c (M_r _≈ 23K) and e (M_r _≈ 11K) could only be detected with the human Fc-specific antiserum (Panel B). Band d (M_r _≈ 12K) was only reactive with the kappa chain-specific antiserum (Panel C).

### *N. tabacum *leaf extracts at neutral pH exhibit little proteolytic activity against IgG

**Figure 3:**
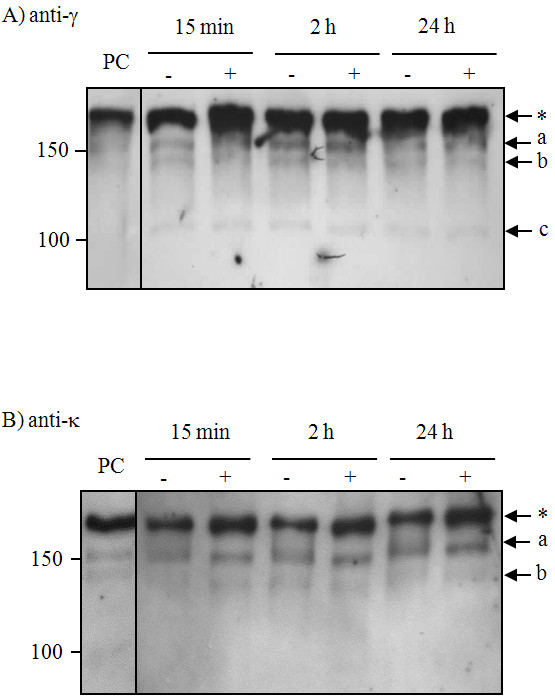
**Western blot analysis of exogenous human IgG_1_κ incubated with wild type *N. tabacum *leaf extract with or without protease inhibitor cocktail**. Leaf discs from wild type *N. tabacum *plants were extracted in either PBS (-) or PBS supplemented with a protease inhibitor cocktail (+) and exogenous human IgG_1_κ (1.8 ng/lane) was added. The mixtures were incubated at room temperature for the indicated times and proteins separated by 6% SDS-PAGE under non-reducing conditions. The proteins were blotted onto nitrocellulose and probed with anti-human γ1 (Fc region specific) antiserum (Panel A) or anti-human κ antiserum (Panel B). The control (PC), is a human IgG_1_κ (1.8 ng/lane). The asterisk indicates the fully assembled 2G12 antibody; lower case letters (a, b and c) indicate antibody fragments.

The presence of the majority of immunoglobulin fragments in plant extracts prepared with protease inhibitor cocktail suggested that proteolytic degradation might occur predominantly *in planta *prior to extraction. In order to investigate the influence of proteases released during the extraction process, we used *Nicotiana tabacum *leaf extracts from non-transgenic plants, prepared in either PBS (pH 7.4) or PBS supplemented with a protease inhibitor cocktail, to which a standard amount of a human IgG_1_κ MAb was added. Samples were incubated for either 15 mins, 2 hrs or 24 hrs at room temperature and analysed by western blotting (Figure 3), using an anti-gamma chain antiserum (Panel A) or anti-kappa chain antiserum (Panel B).

The commercial MAb preparation consists of a predominant single band (labelled with an asterisk) at the expected M_r _of ≈ 180K (Lane PC). The smaller fragments (bands labelled a and b) were also evident in both anti-heavy and anti-light chain blots.

**Figure 4:**
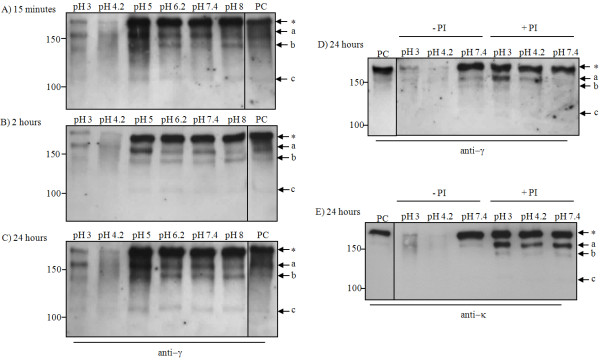
**Western blot analysis of protease-activity in wild type *N. tabacum *leaf extracts of different pH**. Human IgG_1_κ was mixed with wild-type *N. tabacum *leaf extracts buffered at pH 3.0, 4.2, 5.0, 6.2, 7.4 or 8.0 and incubated at room temperature for 15 mins (Panel A); 2 hrs (Panel B); or 24 hrs (Panel C). Proteins were separated by 6% SDS-PAGE under non-reducing conditions. The proteins were blotted onto nitrocellulose and probed with anti-human γ1 (Fc region specific) antiserum. PC is the positive control human IgG_1_κ (1.8 ng/lane). For three of the pH conditions, the effect of a protease inhibitor cocktail was tested (± PI, Panels D and E). Samples were tested at 24 hrs and immunodetection was with anti-heavy chain antiserum (Panel D) or anti-light chain antiserum (Panel E). The asterisk indicates the fully assembled 2G12 antibody; lower case letters (a, b and c) indicate antibody fragments.

This pattern of bands was not significantly affected by incubation with the tobacco extract for up to 24 hrs. The intensities of these bands did not vary over time. Moreover, the addition of the cocktail of protease inhibitors had no significant effect at any of the time points. A faint band (M_r _≈ 120K, labelled c) was observed on some blots due to specific blotting conditions. This band is unlikely to be the result of proteolytic degradation in the plant extract as it is also present in the positive control lanes in Figure 4 Panels A-C.

### IgG proteolysis by tobacco plant extracts is activated at acidic pH

In the next experiment, the influence of extraction buffer pH on the proteolytic activity of *N. tabacum *leaf extracts was investigated. Leaf tissue was extracted in buffers at different pH and incubated at room temperature with exogenous human IgG. Samples were taken after 15 mins, 2 hrs or 24 hrs and analysed by SDS-PAGE and western blotting using an anti-gamma chain antiserum (Figure 4, Panels A-C). The results showed that at pH > 6, there was no significant change in the intensity of the intact IgG (labelled with an asterisk). The smaller bands a, b and c (all present this time in the control sample) did not alter in intensity with decreasing pH. This is true for all the time points investigated (Figure 4, Panels A-C). At pH 5, the intensity of band a increased at all three time points. When the pH was reduced further to 4.2 or 3, a dramatic loss of immunoreactive material occurred, even at the 15 mins time point.

In order to verify whether pHs < 5 might be responsible for the activation of proteases present in *N. tabacum *leaf extracts, we compared IgG samples that were incubated with plant extracts at pH 7.4, pH 4.2 or pH 3, in the presence or absence of a protease inhibitor cocktail. Samples were incubated for 24 hrs and analysed by western blotting using an anti-human Fc antiserum (Figure 4, Panel D) or anti-human light chain antiserum (Figure 4, Panel E). In the absence of protease inhibitors, no significant degradation was seen at pH 7.4, but extensive antibody degradation was observed at pH 4.2 and pH 3, as before. However, addition of the protease inhibitor cocktail almost completely abrogated the antibody loss at low pH.

**Figure 5:**
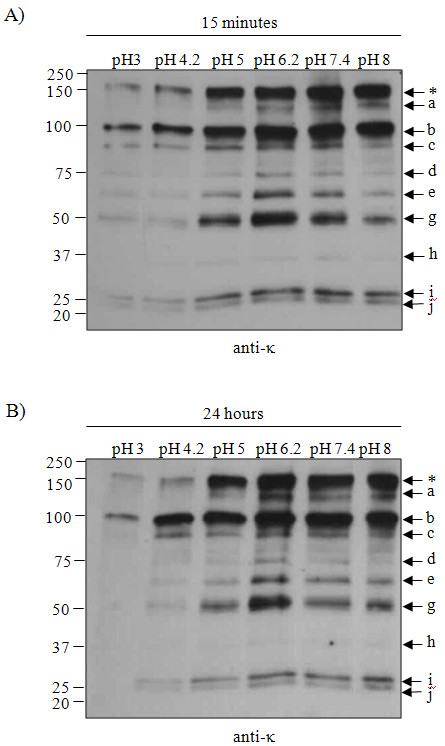
**Western blot analysis of recombinant MAb 2G12 extracted from transgenic *N. tabacum *in different pH-buffers**. Leaf discs from MAb 2G12-expressing plants were extracted in solutions buffered to; pH 3.0, pH 4.2, pH 5.0, pH 6.2, pH 7.4, or pH 8.0. The mixtures were incubated at room temperature for 15 mins (Panel A) or 24 hrs (Panel B). Proteins were separated by SDS-PAGE under non-reducing conditions. Proteins were blotted onto nitrocellulose membranes and probed with anti-human κ antiserum. The asterisk corresponds to the fully assembled 2G12 antibody, while the lower case letters (a to j) indicate antibody fragments.

As pH affected the proteolytic activity of tobacco leaf extracts on exogenous IgG, the effect of extraction buffer pH on MAb 2G12 expressed in transgenic plants was examined. Leaf discs were extracted in different pH buffers as before and incubated for 15 mins or 24 hrs at room temperature. Samples were analysed by SDS-PAGE and western blotting. Immunoreactive bands were detected with anti-kappa antiserum (Figure 5). Recombinant antibody extracted under these conditions was already extensively degraded, as previously observed in Figure 1, but between pH 5-8, there was little difference between the analysed samples, either after 15 mins (Figure 5, Panel A) or 24 hrs incubation (Figure 5, Panel B). At pH 4.2 and pH 3, there was extensive degradation of the antibody. Interestingly, the loss of intact IgG is not associated with the appearance of new degradation bands or increased intensity of existing degradation products, rather it appears that there is complete loss of immunoreactivity of all bands.

### Analysis of MAb 2G12 fragments and secretion in transgenic plant cells

**Figure 6:**
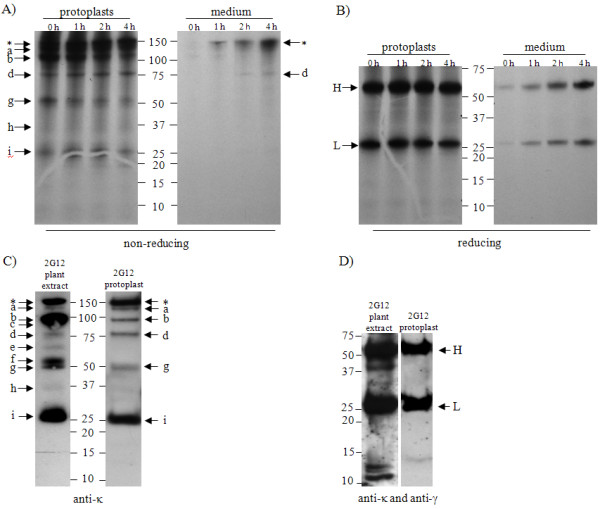
**Pulse chase analysis of antibody fragments in MAb 2G12 transgenic plant cells**. Protoplasts from leaves of transgenic tobacco plants expressing the MAb 2G12 were washed and analysed by SDS-PAGE and western blotting (Panels C and D) or pulse-labelled for 1 hr and chased for the indicated periods of time (Panels A and B). Extracellular medium samples and homogenised cells were subjected to immunoprecipitation with Protein G/Protein A mix. Immunoprecipitated proteins were analyzed by SDS-PAGE and radiography. The asterisk corresponds to the fully assembled 2G12 antibody, while the lower case letters (a to i) indicate antibody fragments. Panels A and C were performed under non-reducing conditions, whereas samples in Panels B and D were reduced with the addition of 5% β-mercaptoethanol prior to boiling.

Protoplasts prepared from MAb 2G12 transgenic plants were pulse-labelled for 30 mins, and subsequently chased following the addition of cold amino acids over a period of 4 hours. During the chase, samples were taken at 0 hrs, 1 hour, 2 hrs and 4 hrs, immunoprecipitated and visualised by SDS-PAGE and auto-radiography to investigate secretion and degradation of MAb 2G12 under non-reducing conditions (Figure 6, Panel A) and reducing conditions (Figure 6, Panel B). Intact MAb 2G12 (indicated with an asterisk) was identified in the protoplasts at 0 hrs. The amount of intact MAb diminished with time inside the protoplasts, concomitant with a gradual increase in full length MAb in the medium. This indicates efficient secretion of MAb 2G12, as has been described previously for another MAb [25]. The characteristic pattern of smaller species (a, b, d, g, h and i) was observed in the cellular samples. Three of these bands (bands a, b and g) decreased in intensity over time, whereas others seemed to be stable within the cell (bands d, h and i). The result suggests that fully assembled MAb 2G12 is efficiently secreted from transgenic plant cells, while smaller immunoreactive species of MAb 2G12 are present intracellularly, remain in the cell and are not secreted, with the exception of band d which was visible after 2 hrs chase in the medium. Under reducing conditions (Panel B) free heavy chain (indicated H, M_r _≈ 55K) and free light chain (indicated L, M_r _≈ 25K) were the only bands detected by radiolabelling.

To establish the pattern of antibody bands within the cells under steady state conditions, unlabelled protoplasts were prepared from leaf tissue of two month old transgenic plants expressing MAb 2G12 and analysed by SDS-PAGE and western blotting under non-reducing (Panel C) and reducing conditions (Panel D). For each condition, these results were compared to the pattern obtained from a crude extract of the same leaf material. At steady state, free heavy or light chain were again the only bands detected under reducing conditions within the cells, whereas several additional fragments could be observed in the crude extract. The presence of bands that did not correspond to assembly intermediates was also apparent in the non-reduced crude sample (Panel C).

## Discussion

Many recombinant MAbs extracted from transgenic plants demonstrate a characteristic and reproducible pattern of fragmentation. Such a pattern was observed in the MAb 2G12 plants studied here. Despite findings which show that recombinant protein degradation occurs *in vivo *[25,26], the possibility that degradation fragments are formed as a result of proteases that are activated during plant cell and tissue disruption cannot be excluded [14]. As a consequence, it is routine practice to include a range of protease inhibitors in extraction buffers. However, the efficacy of protease inhibitors has never been proven, furthermore they are frequently expensive and unlikely to be practical for commercial scale up.

The identity of the IgG fragments has also not yet been determined. Whilst some may represent assembly intermediates of IgG (e.g., free heavy or light chains, H/L heterodimers or H/H homodimers), these cannot account for all of the fragments observed. Indeed, when analysed under reducing conditions (Figure 2), fragments with a lower molecular weight than the free heavy and free light chains were observed and these can only be due to degradation. Based on the data presented, tentative assignments have been made for each of the bands observed in Figure 1B. Species which correspond to bands present in the protoplast preparation (Figure 6, Panel D) are likely to be assembly intermediates (bands a, b, d, g and i in both figures), as no other fragments were observed in protoplasts under reducing conditions. Band g shares a molecular weight (M_r _50K) with a species previously described as a degradation fragment (Fab-like fragment) [3,5,27]. This possibility cannot be excluded as the predicted mobility of both components of a reduced Fab-like fragment matches that of the light chain. Bands c, e, f and h in Figure 1B were present only in the plant tissue extract (Figure 6, Panel C), and are likely to originate in the apoplastic space as they were not affected by the addition of protease inhibitor (Figure 1, Panel B) and are not apparent in the protoplast preparation (Figure 6, Panel C).

The loss of plant-expressed antibodies due to proteolytic degradation *ex planta *and *in planta *is a major concern regarding stability, final yield of plant-expressed MAbs and validation of purification. Development of strategies to prevent proteolytic degradation of plant-expressed antibodies would therefore be a worthwhile consideration, so it is important to understand when and where this degradation occurs.

Non-transgenic *N. tabacum *leaf extracts have little or no anti-IgG proteolytic activity in the pH range commonly used for experimental extraction. In addition, the use of a broad acting cocktail of protease inhibitors at high concentration had little effect on the IgG fragment pattern for MAb 2G12. These findings suggest that degradation of MAb 2G12 is not related to the process of extraction, more likely, degradation occurs before extraction, and either along the secretory pathway, in the apoplastic space, or in subcellular storage compartments [28]. These observations support the findings of Hassan *et al. *[29] who showed little benefit from using protease inhibitors during the extraction of a murine IgG_1_κ at neutral pH to prevent the loss of functional MAb. Endoproteases that can degrade monoclonal antibodies are undoubtedly released during the extraction process [17,30,31], but significant proteolytic activity was only observed here at pH < 5 and in this instance, degradation could be countered by the protease inhibitor cocktail.

At low pH, degradation of IgG is characterised by a complete loss of MAb, rather than the appearance of new, smaller species. An explanation for this phenomenon could be that plant proteases active in acidic conditions (e.g. aspartic acid proteases) may show extensive activity on antibody Fc regions, degrading it to smaller peptides [32,33]. Kim *et al. *[34] suggested that under acidic conditions (pH 3.9) a conformational change in the Fc region of an IgG takes place, which makes the antibody more accessible to proteases.

The organelles of the early secretory pathway are the sites of initial polypeptide folding and maturation. Given these roles, the luminal compartments of these organelles are not widely recognised as possessing broad-spectrum proteolytic activity. The established paradigm for 'quality control' in the ER consists of prolonged association with ER-resident chaperones, N-glycan modification, retrotranslocation and complete degradation by the proteasome in the cytosol [35,36]. Alternative routes for luminal disposal of proteins that fail to mature have been mooted, either through trafficking in a complex with BiP to the vacuole [37] or via an ER subdomain rich in proteases [38]. It is important to remember that antibodies have not evolved in the context of the plant protease degradome, and they could therefore represent novel targets for plant proteases [14]. However, previous studies on transgenic tobacco tissue have shown that by blocking early transport events in the secretory pathway, and thereby causing an accumulation of antibody in an ER-Golgi super-compartment, the accumulation of intact mouse IgG_1 _MAb could in fact be markedly improved [2]. High levels of intact recombinant protein have been reported to accumulate in ER-derived compartments, such as protein bodies [39].

Here, we have shown that IgG-expressing protoplasts do not accumulate MAb proteolytic fragments when analysed at steady state. Furthermore, a discrete pulse of expression analysed over a 4-hour chase period revealed no species that did not correspond to the predicted mobility of an IgG assembly intermediate. Hence, it would appear unlikely that discrete intracellular proteolytic fragments are not detectable at steady state due to rapid clearance through further degradation. In similar pulse-chase experiments, Hadlington *et al. *describe the formation of fragments characteristic of vacuolar proteolysis when a hybrid murine MAb with both gamma and alpha constant regions was expressed in protoplasts [40]. These fragments were not observed in our experiments. Over the course of the chase, several smaller species are lost from the protoplasts apparently without fragmentation or secretion into the medium, which suggests that a significant proportion of IgG tetramers undergo a delayed assembly. The cause of this slow assembly is unclear, although it has been reported that an excess of light chain may be required for efficient release of heavy chain from the heat shock protein 70 (HSP70) family chaperone BiP [41], and no attempt was made here to optimise the stoichiometry of the components.

In contrast to the secretory pathway, the apoplast is a likely compartment for the formation and accumulation of the proteolytic fragments observed in these experiments. The pH of apoplastic fluid is between pH 5-6 [42]. The apoplastic space is regarded to be rich in acidic proteases [6,7] and a number of candidate proteases have been identified. Secretion of six proteases into the leaf apoplastic space of different plant species (tomato, *Arabidopsis*, *N. benthamiana*), belonging to the cysteine protease (papain-like), aspartic protease (pepsin-like) and serine protease (subtilisin-like and carboxypeptidase-like) families has been reported [10]. Seventeen peptides corresponding to predicted plant aspartic, cysteine, and serine proteases were identified from zymography of intercellular fluid from *N. tabacum *[43]. A plasma membrane associated metalloproteinase has recently been reported in tobacco cells [44], and a member of the legumain Asn-specific cysteine endopeptidase family has also been described in the plant cell wall [45].

## Conclusions

We conclude that the fragmentation pattern of heterologously expressed MAb in *N. tabacum *is not primarily due to proteolysis arising during extraction unless acidic extraction buffers are used. Instead, the fragmentation pattern observed when samples are subjected to SDS-PAGE under reducing conditions appears to reflect *in planta *proteolysis, and this activity was restricted to the apoplast rather than the cells. If this is the case, it is unlikely that any extraction strategy that utilises exogenous measures to control proteolytic degradation will have a significant effect. Measures aimed at preventing degradation of IgG within the apoplastic space have the potential to increase yield and homogeneity of this important class of molecule and need to be explored further.

## Methods

### Plant material

*Nicotiana tabacum (var. xanthi) *plants (2-3 months old) were used. They were either wild-type (non-transgenic) or transgenic and homozygous for the γ and κ chains of the human IgG_1_κ MAb 2G12 (kindly provided by Thomas Rademacher and Eva Stöger). For each sample, a single leaf disc (average mass = 7.56 mg) was excised from a young leaf using the lid of a 1.5 ml Eppendorf tube as a punch. Leaf discs were homogenised using a plastic pestle in a 1.5 ml microcentrifuge tube and incubated with 300 μl of the appropriate extraction buffer.

### Antibody stability in crude plant extracts

A range of extraction buffers at different pHs were used for the extraction. These were phosphate buffered saline (PBS) at pH 7.4; 0.1 *M *citric acid-phosphate buffer at pH 3.0, 4.2, 5.0, or 6.2, or 50 mM Tris-HCl buffer at pH 8.0. In some cases, where indicated, the extraction buffer was supplemented with 7x Complete^® ^Protease Inhibitor Cocktail (Roche, UK) and 7 mg/ml pepstatin A (Sigma, UK).

Extracted samples were centrifuged for 5 mins at 17 000 × g and the supernatant collected.

For experiments involving supplemented (exogenous) antibody, a human IgG_1_κ MAb (The Binding Site, UK) was added to wild type plant extracts to a final concentration of 0.2 ng/μl. Samples were incubated for 15 mins, 2 hrs or 24 hrs at room temperature, before being analysed by SDS polyacrylamide gel electrophoresis (SDS-PAGE) and western blotting.

### Protein purification

Transgenic *N. tabacum *plants expressing 2G12 MAb were ground in PBS (pH 7.4). The homogenate was centrifuged for 30 mins at 15 000 × *g*, 10°C, the supernatant filtered through No. 3 Whatman paper, the pH adjusted to 7.6 and incubated on ice for 30 mins. The sample was centrifuged again (17 0000 × g, 30 mins, 10°C) and the supernatant was filtered (0.22 μm, Millipore) and loaded onto a mixed Protein G-Sepharose (Sigma, P3296)/Protein A-Agarose (Sigma, P3476) column. The column was washed with PBS, antibody eluted with 100 mM glycine (pH 2.5) and the pH of the eluate neutralised with 1 M Tris base (pH unadjusted).

### SDS-PAGE and western blotting

Protein samples were separated on either 15% polyacrylamide gels under reducing conditions, 6% polyacrylamide gels or 4-12% Bis-Tris gradient gels (Invitrogen) under non-reducing conditions. Aliquots (60 μl) of the processed samples were boiled for 3 mins with 15 μl of 5x SDS-PAGE sample buffer, (50% (v/v) glycerol, 10% (v/v) sodium dodecylsulfate, 0.3125 M Tris-HCl (pH 6.8) and 0.125% (w/v) bromophenol blue) for analysis under non-reducing conditions, or supplemented with β-mercaptoethanol to 5% (v/v) for analysis under reducing conditions. As a control in the western blotting, a human IgG_1_κ MAb (The Binding Site, UK) was loaded at either 1 ng/lane or 1.8 ng/lane. Separated proteins were blotted onto nitrocellulose membranes using a semi-dry transfer device (BioRad). The membranes were incubated with 5% (w/v) non-fat milk powder in TBS for at least 30 mins to block free protein binding sites. A polyclonal HRP-labelled goat anti-human IgG (Fc fragment) antiserum (Jackson ImmunoResearch, 109-035-008) was used for detection of the heavy chain. Detection of the light chain was performed with an HRP-labelled anti-human kappa (κ) reagent (Sigma, A7164). Blots were washed five times with PBS containing 0.2% (v/v) Tween 20 and developed using the ECL Plus western blotting detection system (GE Healthcare).

For Coomassie staining, Protein A/G purified plant samples were separated on SDS-acrylamide gels under reducing conditions (5x SDS-PAGE sample buffer supplemented with 5% (v/v) 2-mercaptoethanol) and incubated for 1 hr with Coomassie InstantBlue (Expedeon). A human IgG_1_κ MAb (The Binding Site, UK), used as a positive control (PC), was loaded at 500 ng/lane.

### Preparation of protoplasts and *in vivo *labelling

Leaves from 4-6 week old transgenic *N. tabacum *plants expressing MAb 2G12 were used for the preparation of protoplasts as described in Pedrazzini [46] and incubated for 16 hrs in 4% (w/v) Cellulase Onozuka R-10, 2% (w/v) Macerozyme Onozuka R-10 (Apollo Scientific), in K3 medium (Gamborg's B5 basal medium with minimal organics, 3.78 g/L; supplemented with 750 mg/L CaCl_2_.2H_2_O; 250 mg/L NH_4_NO_3_; 136.2 g/L sucrose; 250 mg/L xylose; 1 mg/L 6-benzylaminopurine; and 1 mg/L α-naphthalene acetic acid, pH 5.5) at 25°C in the dark. Tissue disruption and recovery of viable protoplasts was in K3 medium. The radiolabelling period (50 μCi/ml Expre^35^S^35^S cell labeling mix (73% L-[^35^S]methionine and 22% L-[^35^S]cysteine; Perkin Elmer, Beaconsfield, UK) was for 30 mins and the chase was started with the addition of excess cold amino acids (time point 0 h). Samples were taken at 0 hours, 1 hour, 2 hours and 4 hours after labelling and protoplast were precipitated by the addition of three volumes of W5 medium (154 mM NaCl, 5 mM KCl, 125 mM CaCl_2_.2H_2_O, and 5 mM glucose). Samples were homogenized in homogenisation buffer (150 mM Tris-HCl, pH 7.5; 150 mM NaCl; 1.5% (v/v) Triton X-100) or 2 volumes of the same buffer containing 0.25% (w/v) gelatin, respectively; supplemented with one tablet of Complete Protease Inhibitor Cocktail (Roche) per 15 ml). Immunoprecipitation of labeled samples was performed as described in Pedrazzini [46] with a mix of Protein A-Agarose beads (Sigma) and Protein G-Sepharose beads (Sigma) in NET buffer (50 mM Tris-HCl, pH 7.5; 150 mM NaCl; 1 mM EDTA; 0.1% (v/v) Nonidet P-40). After removal of the supernatant, immunoprecitated polypeptides were either stored frozen at -20°C or immediately analysed by reducing or non-reducing SDS-PAGE on 4-15% gradient gels. Gels were fixed for 30 mins in 10% (v/v) acetic acid, 40% (v/v) ethanol and amplified for 20 mins using NAMP100 (GE Healthcare), followed by autoradiography.

## List of abbreviations

Fc: Fragment crystallizable; HRP: horseradish peroxidase; hrs: hours; IgG: Immunoglobulin G; MAb: Monoclonal antibody; mins: minutes; PBS: phosphate-buffered saline; M_r_: relative molecular mass; SDS-PAGE: sodium dodecylsulfate-polyacrylamide gel electrophoresis; TBS: Tris-buffered saline.

## Authors' contributions

VH carried out the experimental work and wrote the manuscript. CvD supervised the project. CvD, JM and PD planned the project and helped to draft the manuscript. MP helped with the pulse chase experiment. All authors read and approved the final manuscript.
